# Status and perceptions of ChatGPT utilization among medical students: a survey-based study

**DOI:** 10.1186/s12909-025-07438-7

**Published:** 2025-06-04

**Authors:** Na Hu, Xiao Qin Jiang, Yi Da Wang, Yan Ming Kang, Zhen Xia, Hao Hui Chen, Sai Nan Duan, Dong Xu Chen

**Affiliations:** 1 Department of Anesthesiology, West China Second University Hospital, Sichuan University, Chengdu, China; 2 Key Laboratory of Birth Deficits and Related Diseases of Women and Children, Sichuan University, Ministry of Education, Chengdu, China; 3 Department of Anesthesiology, Chengdu Hi-Tech Zone Hospital for Women and Children, Chengdu, 610041 China; 4 Key Laboratory of BioResource and Eco-Environment of Ministry of Education, College of Life Science, Sichuan University, Chengdu, China; 5 West China School of Medicine, Sichuan University, Chengdu, China

**Keywords:** Artificial intelligence, ChatGPT, Medical education, Medical students

## Abstract

**Background:**

The integration of ChatGPT with educational settings is happening at an unprecedented rate, and there is a growing trend for students to use ChatGPT for various academic work. Although numerous studies have evaluated the knowledge, attitudes, and practices related to ChatGPT among students in diverse medical fields, there remains a notable absence of such research within the Chinese context.

**Methods:**

The questionnaire survey was conducted to a sample of 1,133 medical students from various medical colleges across Sichuan Province, China, between May 2024 and November 2024 to explore the awareness and attitudes of medical students towards ChatGPT. Descriptive statistics were used to tabulate the frequency of each variable. A chi-square test and multiple regression analysis were employed to investigate the factors influencing participants’ positive attitudes toward the prospective use of ChatGPT.

**Results:**

The findings revealed that 62.9% of participants had employed ChatGPT in their medical studies, with 16.5% having utilized the tool in a published article. Participants primarily used ChatGPT for searching information (84.4%) and completing academic assignments (60.4%). However, concerns were expressed regarding the potential for ChatGPT to disseminate misinformation (76.9%) and facilitate plagiarism or complicate its detection (65.4%). Despite these concerns, 64.4% of respondents indicated a willingness to use ChatGPT to seek assistance with learning problems. Overall, a majority of participants (60.7%) maintained a positive attitude on the future use of ChatGPT in the medical field.

**Conclusion:**

Our research showed that while most medical students perceived ChatGPT as a valuable tool for academic study and research, they remained cautious about its potential risks, particularly regarding misinformation and plagiarism concerns. Despite these reservations, a majority participants indicated a willingness to incorporate ChatGPT into their academic workflow, specifically for problem-solving tasks, and maintained optimistic perspectives regarding its potential integration into medical education and clinical practice. It is therefore essential to improve student literacy about AI, develop clear guidelines for its acceptable use, and implement support systems to ensure that medical students are fully prepared for the upcoming integration of AI into medical education.

**Trial registration:**

Not applicable.

**Supplementary Information:**

The online version contains supplementary material available at 10.1186/s12909-025-07438-7.

## Introduction

Chat Generative Pretrained Transformer (ChatGPT) is a revolutionary artificial intelligence (AI)-based language model that utilizes deep learning techniques to generate human-like responses to natural language inputs [1]. Upon its release, ChatGPT garnered significant attention across society and has been extensively adopted across diverse sectors, such as software development, journalism, literary creation, and other domains, demonstrating its versatility [2, 3]. Furthermore, it has exhibited distinct value within the medical field by facilitating novel interaction modalities between patients and healthcare providers, as well as supporting medical decision-making, education, research, and additional areas [4].

In the domain of medical education, ChatGPT has exhibited its capacity to synthesize and utilize foundational medical knowledge effectively. Without any formal training or reinforcement, it is capable of meeting or nearing the passing threshold in all three exams of the United States Medical Licensing Examination (USMLE), achieving a performance level comparable to that of third-year medical students. Moreover, this tool demonstrated a high level of concordance and insight in its explanations. These findings indicated that ChatGPT could play a role in medical education and potentially aid in clinical decision-making [5, 6].

Although, ChatGPT offers a personalized learning experience for medical students and residents, providing a secure environment with immediate feedback [7]. Nonetheless, ChatGPT is met with skepticism due to concerns about potential inaccuracies and ethical issues, such as the risks of bias, plagiarism, and copyright infringement [8, 9]. Therefore, it is imperative to conduct empirical investigations into the capabilities of this technology as a catalyst for transformative innovation, as well as to assess its associated risks.

The integration of ChatGPT with educational settings is happening at an unprecedented rate, and there is a growing trend for students to use ChatGPT for various academic work [10]. This study represents one of the first systematic investigations into the awareness, usage patterns, and perceptions of ChatGPT among medical students in China. While prior research has explored AI adoption in medical education globally [11–13], the unique academic, cultural, and regulatory context of China—where AI integration faces distinct opportunities and challenges—remains under examined [14]. This study will fill this critical gap by providing empirical data on how Chinese medical students engage with ChatGPT, their concerns and optimism toward AI’s future role in medicine. This regional perspective contributes original insights to the global discourse on AI in medical education. Therefore, the main objective of this cross-sectional study was to evaluate the perceptions of medical students regarding ChatGPT, conduct an in-depth analysis of their usage behavior characteristics, and systematically investigate their attitudinal dispositions towards this technology.

## Methods

### Design

Data from survey respondents remained anonymous, with no personal information collected. The study was deemed exempt research by the West China Second Hospital of Sichuan University Institutional Review Board (Approval No. 2024-083, Date: June 12, 2024) and used password-protected survey platform access for data security. The survey focused on Sichuan province-based medical students. Survey participation was voluntary and uncompensated.

This study adopted convenience sampling method and was conducted from May 2024 to November 2024 across medical colleges in Sichuan Province, China. Participants scanned a QR code to enter an introduction page and voluntarily participate in the survey. The introduction section of the survey explicitly addressed the following points: (1) assurance of confidentiality and anonymity of responses; and (2) affirmation of voluntary participation in the survey. Subsequently, participants were required to complete an informed consent form, which stated, “You acknowledge and agree that the data collected may be used for research analysis,” as a prerequisite for proceeding with the survey. Given the exploratory nature of this study and the need for rapid data collection amid evolving ChatGPT adoption trends, convenience sampling allowed efficient access to medical students within targeted universities in Sichuan Province. Despite convenience sampling, participants were recruited from multiple medical schools and year groups to capture diverse perspectives. While convenience sampling may limit generalizability, it enabled timely assessment of ChatGPT’s real-world use patterns among Chinese medical students—a population underrepresented in existing literature.

### Design and content of the questionnaire

The questionnaire (Table S1) was developed based on a comprehensive review of the existing literature on related topics and was modified according to local perceptions in China [3, 12, 13, 15]. Before the formal investigation, a pilot test of questionnaires was conducted in this study. Six experts were invited for systematic content validity evaluation (Table S2). The Item-Content Validity Index (I-CVI) of all items was ≥ 0.80, and the Scale-Content Validity Index/Average (S-CVI/Ave) was ≥ 0.90. And based on expert suggestions, the usage purposes of ChatGPT are divided into three fields: work assistance, academic research, and clinical application. Subsequently, 10 students were recruited from different clinical disciplines, maintaining a male-to-female ratio of 1:1. The participants included undergraduates, postgraduates, and PhD. These medical students completed the questionnaire, recorded the time taken for completion, and identified any issues encountered. Based on their feedback, the questionnaire was further refined and specific questions addressing the challenges faced in using ChatGPT were incorporated. It should be noted that the responses from the pilot test were excluded from the final data analysis.

The finalized version of the questionnaire, comprising 20 questions organized into four sections, was published on the WJX platform (https://www.wjx.cn/). These sections addressed the demographic characteristics of the participants, the engagement in academic research, the usage of ChatGPT, and the attitudes towards the application of ChatGPT in the medical field. Participants were queried regarding their prior use of ChatGPT. Respondents who affirmed their usage proceeded to complete the questionnaire (question 9), whereas those who negated were prompted to select their reasons for not utilizing ChatGPT and then ended the questionnaire (question 11).

### Conduct of the survey

The questionnaire was distributed through the popular social media application WeChat (Tencent, Inc., Shenzhen, China). According to data from Tencent, the global monthly active users of WeChat have exceeded 1 billion. Moreover, the WeChat platform has been used to develop other functions. Our questionnaire was distributed through WeChat groups. The team members contacted doctors and university teachers from various medical schools, including affiliated hospitals, through WeChat, informed them of the purpose of the survey, and then forwarded the questionnaire to them in WeChat groups of students of different grades and majors. Reminder WeChat were sent to eligible nonrespondents, with a maximum of 3 reminders.

### Data analysis

After collecting questionnaires to the greatest extent possible, data cleaning was conducted. The questionnaires were screened based on the following predetermined criteria: (1) responses with excessive missing data, accounting for two-thirds of the total questions; (2) responses exhibiting uniform or highly regular answer selections; and (3) all questionnaires with less than 60 s to complete the answers were excluded; and if it takes less than 30 s to complete the questionnaire after answering the 11 question, it will be excluded. The aforementioned time thresholds were established as reasonable response times based on a pilot test conducted by 10 medical students from our research team.

The responses from all participants were extracted from the WJX platform and subsequently imported into Excel for further analysis using SPSS software (version SPSS 27). Participants’ attitudes towards the prospective use of ChatGPT in academic research were categorized as follows: those who opted to maintain their current frequency of use or learn more deeply and use more widely were classified as having a positive outlook; those who preferred to observe the usage patterns among scholars, adapt based on trends in published literature and future academic requirement were deemed to have a neutral stance; and those who chose to discontinue use were categorized as having a negative perception. Descriptive statistics were used to tabulate the frequency of each variable. The analysis of factors associated with participants’ positive attitudes towards future ChatGPT use was conducted using the Chi-square test and multiple regression analysis. *P*<0.05 indicated a significant difference between the variables.

## Result

### Demographics

The survey comprised a total of 1,133 participants, of whom 432 were male (38.1%) and 701 were female (61.9%) (Table 1). A significant proportion of the participants held a bachelor’s degree (53.8%), while 10.1% had attained a doctoral degree. A majority of the respondents were majoring in clinical medicine (55.7%), and their primary area of research was clinical research (54.3%).

**Table 1:** Participant demographics

Variable	Total (*n* = 1133)
Sex, No. (%)	
Male	432 (38.1)
Female	701 (61.9)
Education level, No. (%)	
Junior college and below	108 (9.5)
Undergraduate	609 (53.8)
Postgraduate	302 (26.7)
PhD	114 (10.1)
Major, No. (%)	
Basic Medicine	41 (3.6)
Clinical Medicine	631 (55.7)
Stomatology	162 (14.3)
Public Health and Preventive Medicine	19 (1.7)
Traditional Chinese Medicine	6 (0.5)
Integrated Chinese and Western Medicine	8 (0.7)
Pharmacy	43 (3.8)
Chinese Medicine	4 (0.4)
Forensic Medicine	3 (0.3)
Medical Technology	37 (3.3)
Nursing	49 (4.3)
Biomedical Engineering	70 (6.2)
Acupuncture	2 (0.2)
Others	58 (5.1)
Main research area, No. (%)	
Clinical research	615 (54.3)
Basic research	299 (26.4)
Education and popular science	18 (1.6)
Others	201 (17.7)

**Table 2:** Factors related to attitude toward the use of ChatGPT in medical research in the future

	What is your attitude towards using ChatGPT for academic research in the future?					Multivariate analysis	
Variable	Positive (*n* = 433)	Neutral (*n* = 277)	Negative (*n* = 3)	Cramer’s V	*P* value^*^	Estimate and 95% Confidence interval	*P* value^#^
Sex, No. (%)				0.143	<0.001		
Female	219 (54.9)	177 (44.4)	3 (0.8)			Reference	
Male	214 (68.2)	100 (31.8)	0 (0.0)			1.69 (1.22, 2.35)	0.003
Education level, No. (%)				0.113	0.005		
Junior college and below	25 (51.8)	18 (41.9)	0 (100.0)			Reference	
Undergraduate	223 (57.2)	166 (42.6)	1 (0.3)			0.72 (0.38, 1.33)	0.378
Postgraduate	121 (62.7)	72 (37.3)	0 (0.0)			0.81 (0.44, 1.13)	0.191
PhD	64 (73.6)	21 (24.1)	2 (2.3)			1.09 (0.79, 1.49)	0.363
Major, No. (%)				0.159	0.092		
Basic Medicine	19 (67.9)	8 (28.6)	1 (3.6)				
Clinical Medicine	266 (64.9)	142 (34.6)	2 (0.5)				
Stomatology	41 (51.2)	39 (48.8)	0 (0.0)				
Public Health and Preventive Medicine	7 (58.3)	5 (41.7)	0 (0.0)				
Traditional Chinese Medicine	2 (100.0)	0 (0.0)	0 (0.0)				
Integrated Chinese and Western Medicine	2 (100.0)	0 (0.0)	0 (0.0)				
Pharmacy	16 (53.3)	14 (46.7)	0 (0.0)				
Chinese Medicine	3 (100.0)	0 (0.0)	0 (0.0)				
Forensic Medicine	0 (0.0)	1 (100.0)	0 (0.0)				
Medical Technology	9 (34.6)	17 (65.4)	0 (0.0)				
Nursing	18 (50.0)	18 (50.0)	0 (0.0)				
Biomedical Engineering	36 (67.9)	17 (32.1)	0 (0.0)				
Acupuncture	0 (0.0)	1 (100.0)	0 (0.0)				
Others	14 (48.3)	15 (51.7)	0 (0.0)				
Main research area, No. (%)				0.056	0.605		
Clinical research	230 (62.0)	140 (37.7)	1 (0.3)				
Basic research	124 (61.1)	77 (37.9)	2 (1.0)				
Education and popular science	9 (69.2)	4 (30.8)	0 (0.0)				
Others	70 (55.6)	56 (44.4)	0 (0.0)				
Have you used ChatGPT in any of the articles you published in the past three years?				0.083	0.086		
Yes	82 (69.5)	36 (30.5)	0 (0.0)				
No	351 (59.0)	241 (40.5)	3 (0.5)				
How often do you use ChatGPT, No. (%)				0.187	<0.001		
Every day	51 (87.9)	6 (10.3)	1 (1.7)			Reference	
Several times a week	126 (72.4)	48 (27.6)	0 (0.0)			4.75 (2.71, 9.00)	< 0.001
Once a week	21 (67.7)	10 (32.3)	0 (0.0)			0.83 (0.43, 1.57)	0.642
Occasionally	185 (54.4)	155 (45.3)	1 (0.3)			1.17 (0.81, 1.74)	0.484
Only once or twice	49 (45.4)	58 (53.7)	1 (0.9)			0.78 (0.42, 1.39)	0.393
How many articles in Chinese have you published, No. (%)				0.219	<0.001		
0	334 (58.9)	233 (41.1)	0 (0.0)				
1–3	87 (68.0)	40 (31.3)	1 (0.8)				
4–6	4 (66.7)	1 (16.7)	1 (16.7)				
7–9	2 (100.0)	0 (0.0)	0 (0.0)				
≥10	6 (60.0)	3 (30.0)	1 (10.0)				
How many articles in English have you published, No. (%)				0.174	<0.001		
0	301 (58.3)	219 (41.6)	1 (0.2)				
1–3	109 (68.6)	50 (31.4)	0 (0.0)				
4–6	11 (68.6)	4 (25.0)	1 (6.3)				
7–9	1 (100.0)	0 (0.0)	0 (0.0)				
≥ 10	5 (50.0)	4 (40.0)	1 (10.0)				

^*^ Chi-square *P* value

^#^ Multivariate analysis was not performed for all variables due to limited sample sizes in certain subgroups. Variables such as major, number of published English papers, and number of published Chinese academic papers were excluded from this analysis

### Knowledge and exposure to ChatGPT

**Fig. 1:**
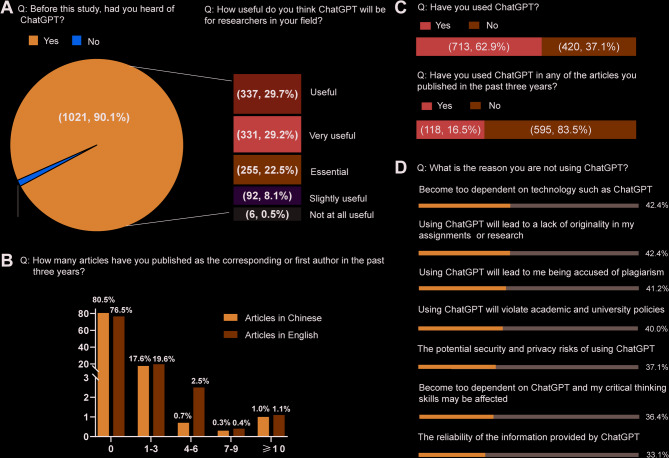
Participants’ research status and knowledge of ChatGPT. (**A**) Knowledge of chatGPT. (**B**) Published articles in the past three years. (**C**) Whether ChatGPT is used in the published articles. (**D**) Summary of reasons not to use ChatGPT

Among the 90.1% of respondents who had heard of ChatGPT, 29.7% considered it useful for researchers in their field (Fig. 1A). Nonetheless, the majority of participants had not published any work in the past three years, with only a minority having done so (Fig. 1B). Even among the 62.9% who used ChatGPT, only 16.5% incorporated the technology into their published articles (Fig. 1C). For the remaining 37.1% of participants who have not used ChatGPT, the primary concerns included dependence on ChatGPT technology (42.4%), issues related to a lack of originality in the assignments or research (42.4%), and allegations of plagiarism (41.2%) (Fig. 1D).

### Status of participants using ChatGPT

**Fig. 2:**
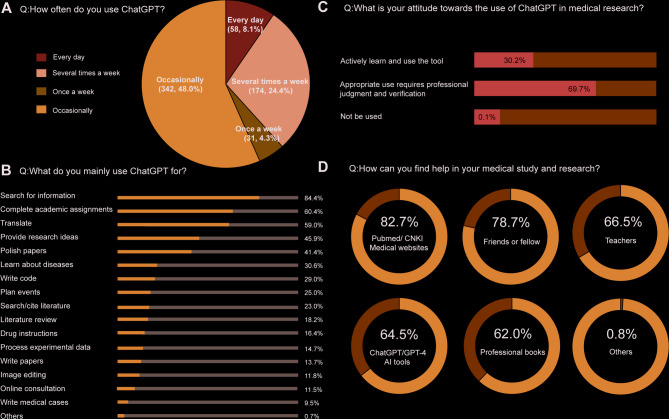
Status of participants using ChatGPT. (**A**) Frequency of ChatGPT. (**B**) Usage of ChatGPT. (**C**) Attitude towards ChatGPT. (**D**) Ways to ask for help in medical study and research

Participates who regularly use ChatGPT at work are still in a minority, with only 8.1% reporting daily usage and 48% indicating occasional use (Fig. 2A). The most prevalent applications included searching for information (84.4%), completing academic assignments (60.4%), translation (59.0%), providing research ideas (45.9%), and polishing papers (41.4%) (Fig. 2B). Despite the enthusiasm surrounding these applications, a majority of respondents (69.7%) expressed a neutral stance, suggesting that while ChatGPT can be utilized, it requires professional judgment and verification (Fig. 2C). Notably, when faced with difficulties in medical studies and research, 64.5% of individuals still opt to utilize AI tools, such as ChatGPT and GPT-4, for support. (Fig. 2D).

### Attitudes towards using ChatGPT in the academic research

**Fig. 3:**
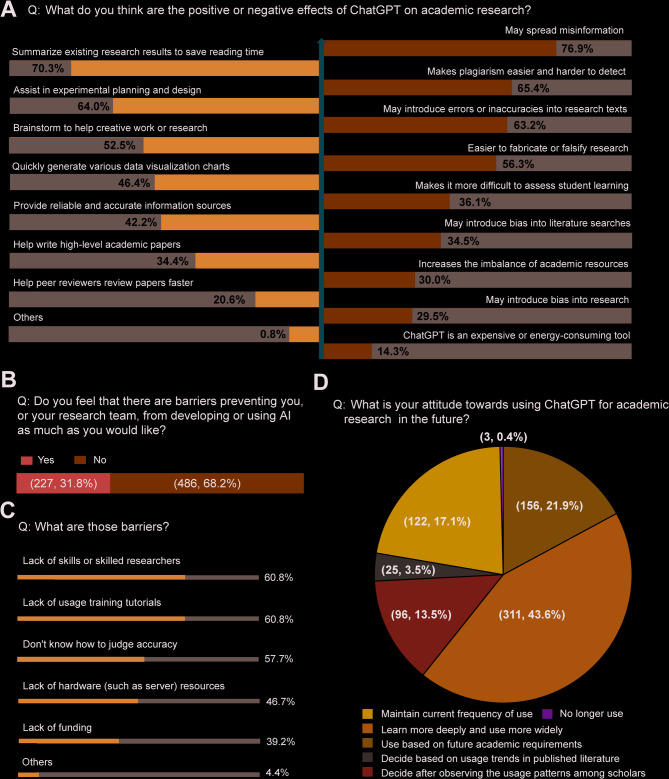
Attitudes towards the application of ChatGPT in the medical field. (**A**) Positive and negative factors of using ChatGPT. (**B**-**C**) Obstacles to the development and use of ChatGPT. (**D**) Attitudes towards future use of ChatGPT for academic research

Respondents acknowledged that ChatGPT could effectively summarize existing research results to save reading time (70.3%), assist in experimental planning and design (64.0%), and brainstorm creative work or research (52.5%). However, concerns have also been raised about the risk of ChatGPT spreading misinformation (76.9%), potentially facilitating plagiarism and complicating its detection (65.4%), falsifying research or disinformation (63.2%) (Fig. 3A). Only 31.8% of respondents reported encountering obstacles in the development or utilization of AI (Fig. 3B), primarily attributed to a shortage of skilled researchers (60.8%) and inadequate availability of training courses (60.8%). Despite its dual nature in the medical field, 60.7% of respondents expressed a positive outlook on future ChatGPT use (Fig. 3D). Notably, no significant link was found between negative impacts and obstacles on its future use among medical students (Table S3).

### Factors associated with attitudes towards using ChatGPT in medical research

Our results indicated that males showed higher positive attitudes compared to females (odds ratio [OR] = 1.69, 95% confidence interval [CI]: 1.22–2.35, *P* = 0.003, Table 2). The positive attitude of high-frequency users (using several times a week) was higher than that of low-frequency users (OR = 4.75, 95% CI: 2.71-9.00, *P* < 0.001).

## Discussion

The findings of this study enhance our comprehension of medical students’ perceptions and attitudes regarding the utilization of ChatGPT within the medical education and research. By employing the Technology Acceptance Model (TAM) and Diffusion of Innovations Theory [16, 17], the observed patterns ChatGPT adoption and usage among medical students can be effectively contextualized. The results indicated that a majority of participants have engaged with ChatGPT in the context of medical research, with some employing it in published research. A substantial proportion of participants expressed a favorable attitude towards the technology. The high adoption rate and positive future outlook align with TAM’s core constructs of perceived usefulness and ease of use, particularly evident in students’ primary utilization for information searching and academic tasks.

Furthermore, the data reveal that male students, high-frequency users are more inclined to possess a favorable attitude towards the technology. The observed gender disparity in ChatGPT adoption may be attributed to sociocultural factors, particularly traditional gender role constructs that influence technological self-efficacy. Male participants typically demonstrate higher levels of confidence in their technical capabilities, potentially stemming from earlier exposure to programming and artificial intelligence curricula [18]. Conversely, female participants exhibited heightened awareness of and concern regarding potential technological risks [19]. Through the Diffusion of Innovations perspective, the gender-based disparities in adoption patterns suggest differential positions along the innovator-laggard continuum, influenced by sociocultural factors rather than educational attainment. Notably, this gender-based divergence persists independent of educational attainment, as evidenced by comparable advanced degree rates between male and female participants (95.9% and 92.5%, respectively). However, the identified concerns about misinformation and plagiarism reflect TAM’s perceived risk component, potentially moderating adoption intentions [20]. These findings suggest that the successful integration of artificial intelligence technologies in medical education necessitates the development of demographically tailored implementation strategies. This theoretical framing underscores the importance of addressing perceived risks and sociocultural barriers while leveraging perceived benefits to promote equitable AI technology integration in medical education, with a particular focus on experiential learning to facilitate positive attitude changes across all user groups [21, 22].

Cross-sectional studies yielded significant insights into the preliminary investigation of ChatGPT’s application in medical education. The majority of initial research concentrated on the perceptions and experiences of students and educators regarding the use of ChatGPT, as well as the determinants influencing students’ intentions to utilize this tool [23]. Our study revealed that 62.9% of participants demonstrated a functional understanding of ChatGPT and utilized it for diverse academic tasks. Notably, 64.5% of respondents expressed a preference for ChatGPT as a primary resource to resolve learning challenges. These results not only reflect ChatGPT’s widespread adoption among medical students but also highlight the critical need for its structured incorporation into medical curricula. This integration can be achieved by embedding the ChatGPT application module into the medical information retrieval course, developing comparative teaching units, demonstrating the synergistic effect of ChatGPT in conjunction with professional medical databases such as UpToDate, and designing clinical thinking training tasks facilitated by ChatGPT. Such measures aim to foster a positive interaction between AI tools and medical education.

Cross-sectional studies, although useful for identifying correlations between variables such as the use of ChatGPT and learning outcomes, are limited in their ability to establish causality. Consequently, numerous randomized controlled trials have been conducted to investigate the integration of AI in medical education. The studies indicated that AI substantially enhanced students’ self-directed learning and critical thinking abilities in comparison to traditional educational methods [24]. When employed as a virtual assistant, AI advanced students’ knowledge and proficiency in surgical skills [25]. Additionally, as an auxiliary medical tool, AI markedly improved the quality and satisfaction associated with clinical decision-making [26]. These existing findings provide valuable insight into the emerging trend of integrating ChatGPT in medical education settings, underscoring the critical role AI now plays in advancing medical education. Other research has demonstrated notable adverse effects, including diminished reporting reliability associated with the use of AI [27, 28]. Considering the mixed outcomes of current research and our findings that medical students utilize ChatGPT to assist with their academic works, there is an equivalent level of concern regarding the potential negative impacts of ChatGPT. Consequently, there remains a necessity for a thorough review and further research to address existing contradictions, alongside the development of guidelines and policies to regulate the ethical use of AI tools like ChatGPT in educational contexts.

In recent years, several universities have incorporated AI into medical education, developing distinctive curriculum systems and gaining valuable experience. Stanford University, for instance, has established an AI laboratory and is actively working to integrate AI technology into clinical practice, thereby creating a premier platform for faculty and students [29]. In Japan, a comprehensive general education curriculum related to AI has been developed for students across all age groups, with an emphasis on fostering interdisciplinary skills. China has systematically accelerated AI education through progressive policy interventions in recent years. The foundational milestone emerged in 2020 when the State Council formally endorsed the integration of artificial intelligence with medical disciplines, signaling strategic alignment between technological innovation and healthcare modernization [30]. Building on this directive, the 2022 national curriculum reform embedded AI fundamentals into compulsory education’s information technology syllabus, catalyzing province-level pilot programs for AI literacy courses in primary and secondary schools [31]. By 2023, this top-down framework had achieved substantial institutional penetration: 498 universities launched dedicated AI undergraduate programs, while 28 state-approved innovation pilot zones were established to bridge theoretical education with industry applications [32]. Collectively, these measures reflect China’s concerted strategy to construct a multi-tiered AI talent ecosystem—spanning K-12 enlightenment, higher education specialization, and applied research incubation—to meet the demands of its digital transformation agenda.

It is important to highlight that in Sichuan Province, high-quality AI medical resources are predominantly concentrated in Chengdu, whereas medical colleges in more remote areas may have limited access, primarily restricted to theoretical instruction. This disparity in resource distribution could result in a structural differentiation in AI literacy among medical students. Furthermore, the “Medicine + AI” interdisciplinary pilot program initiated by Sichuan Province may elevate the baseline competencies of students in certain institutions ahead of schedule [33]. However, this policy advantage is unlikely to be replicated in other provinces that have not implemented the pilot program. the traditional notion in medical education within the province that emphasizes AI as a tool rather than a decision-making subject may suppress students’ critical thinking on the ethical risks of AI, while coastal areas pay more attention to cultivating medical students’ ability to supervise and intervene in AI decision-making. Therefore, as the primary cohort for the prospective implementation of medical AI technology, our investigation into medical students possesses not only regional relevance but also substantial implications for the nationwide digital transformation of medical education. Nonetheless, when extrapolating these findings to other regions, it is essential to adjust them in accordance with the variations in local medical ecosystem.

### Limitation

Several methodological limitations warrant consideration in interpreting our findings. Although convenience sampling facilitated efficient provincial data collection, this approach potentially introduced selection bias, with overrepresentation of participants from tertiary medical institutions possessing advanced educational resources. Consequently, medical colleges in prefecture-level cities, which frequently face substantial constraints in artificial intelligence infrastructure and clinical data accessibility, may be inadequately represented. Furthermore, the study encountered participation challenges among senior medical students engaged in clinical rotations, resulting in potentially reduced response rates from this survey. The AI literacy requirements and utilization patterns among students in non-clinical specialties, including public health and basic medical sciences, remain insufficiently documented, thus constraining the generalizability of our findings across diverse medical education contexts. Additionally, self-selection bias may have influenced our results, as students harboring favorable attitudes toward AI technology might have demonstrated greater propensity to participate in the survey, potentially yielding overly optimistic outcome estimates.

## Conclusion

The potential applications of ChatGPT, as an innovative artificial intelligence tool within the medical domain, are diverse and impactful. Our findings demonstrated that the predominant application of ChatGPT among study participants centers on academic endeavors within medical research, particularly for literature retrieval and scholarly task completion. Multivariate analysis identified significant demographic and behavioral patterns in technology acceptance, with male participants and high-frequency users exhibiting enhanced receptivity toward ChatGPT adoption. These results substantiated the correlation between technological proficiency, research engagement depth, and ChatGPT acceptance rates. Acknowledging the concerns raised regarding potential risks, specifically unauthorized information dissemination and academic integrity violations, we propose three strategic interventions: implementation of comprehensive educational programs to facilitate optimal ChatGPT utilization, establishment of robust guidelines governing ChatGPT application in both academic and clinical domains, and development of an integrated support framework to nurture digital competencies among emerging healthcare professionals.

## Electronic supplementary material

Below is the link to the electronic supplementary material.

Supplementary Material 1

Supplementary Material 2

Supplementary Material 3

## Data Availability

The data presented in this study are available on request from the corresponding author.

## Abbreviations

AI: Artificial intelligence
ChatGPT: Chat Generative Pretrained Transformer
CI: Confidence interval
OR: Odds ratio
I-CVI: Item-Content Validity Index
S-CVI/Ave: Scale-Content Validity Index/Average
TAM: Technology Acceptance Model
USMLE: United States Medical Licensing Exam
