# Supplementary material for: Status and perceptions of ChatGPT utilization among medical students: a survey-based study
Source: BMC Med Educ. 2025 Jun 4;25:831. doi: 10.1186/s12909-025-07438-7 (PMC12135314; doi:10.1186/s12909-025-07438-7)
Supplement: Supplementary file 1 — Supplementary Material 1 [file 12909_2025_7438_MOESM1_ESM.docx]

**Table S1: Questionnaire**

**Informed Consent Item (Mandatory)**

**You acknowledge and agree that the data collected may be used for research analysis?**

- **Yes**
- **No**

1) What’s your gender?

Male

Female

2) What's your level of education?

Junior college and below

Undergraduate

Postgraduate

PhD

3) What is your major?

Basic Medicine

Clinical Medicine

Stomatology

Public Health and Preventive Medicine

Traditional Chinese Medicine

Integrated Chinese and Western Medicine

Pharmacy

Chinese Medicine

Forensic Medicine

Medical Technology

Nursing

Biomedical Engineering

Acupuncture

Others

4) What is your main research area?

Clinical research

Basic research

Education and popular science

Others

5) How many articles in Chinese have you published as the corresponding author or first author in the past three years?

0

1-3

4-6

7-9

≥10

6) How many articles in English have you published as the corresponding author or first author in the past three years?

0

1-3

4-6

7-9

≥10

7) Before this study, had you heard of ChatGPT?

Yes

No

8) How useful do you think ChatGPT will be for researchers in your field?

Essential

Very useful

Useful

Slightly useful

Not at all useful

9) Have you used ChatGPT?

Yes

No

10) Have you used ChatGPT in any of the articles you published in the past three years?

Yes

No

11) What is the reason you are not using ChatGPT?

I am concerned about the reliability of the information provided by ChatGPT

I am concerned that using ChatGPT will lead to me being accused of plagiarism

I am concerned about the potential security and privacy risks of using ChatGPT

I am concerned that using ChatGPT will violate academic and university policies

I am concerned that I may become too dependent on ChatGPT and my critical thinking skills may be affected

I am concerned that I may become too dependent on technology such as ChatGPT

I am concerned that using ChatGPT will lead to a lack of originality in my assignments or research

12) How often do you use ChatGPT?

Every day

Several times a week

Once a week

Occasionally

Only once or twice

13) What is your attitude towards the use of ChatGPT in medical research?

Actively learn and use the tool

Appropriate use requires professional judgment and verification

Not be used

14) What do you mainly use ChatGPT for?

Complete academic assignments

Search for information

Translate

Write code

Plan events

Provide research ideas

Write papers

Polish papers

Process experimental data

Search/cite literature

Literature review

Image editing

Learn about diseases

Write medical cases

Online consultation/assisted diagnosis and treatment

Drug instructions

Others

15) How can you find help in your medical study and research?

Teachers

Friends or fellow help

Professional books

Pubmed/ CNKI/DVE and other medical websites or forums

ChatGPT, GPT-4 and other artificial intelligence tools

16) What do you think are the positive effects of ChatGPT on academic research?

Provide reliable and accurate information sources

Assist in experimental planning and design

Summarize existing research results to save reading time

Help write high-level academic papers

Brainstorm to help creative work or research

Quickly generate various data visualization charts for easy understanding and analysis

Help peer reviewers review papers faster

17) What do you think are the negative effects of ChatGPT on academic research?

May spread misinformation

Makes plagiarism easier and harder to detect

May introduce errors or inaccuracies into research texts (papers, code)

Easier to fabricate or falsify research

May introduce bias into literature searches

Makes it more difficult to assess student learning

May introduce bias into research

Increases the imbalance of academic resources (only scientists from well-resourced universities or companies can be at the forefront)

ChatGPT is an expensive or energy-consuming tool

18) Do you feel that there are barriers preventing you, or your research team, from developing or using AI as much as you would like?

Yes

No

19) What specific obstacles did you or your research team encounter?

Lack of skills or skilled researchers

Lack of usage training tutorials

Don't know how to judge accuracy

Lack of funding

Lack of hardware (such as server) resources

20) What is your attitude towards using ChatGPT for academic research in the future?

Maintain current frequency of use

Learn more deeply and use more widely

Decide after observing the usage patterns among scholars

Decide based on usage trends in published literature

Use based on future academic requirements

No longer use
