# Supplementary material for: Status and perceptions of ChatGPT utilization among medical students: a survey-based study
Source: BMC Med Educ. 2025 Jun 4;25:831. doi: 10.1186/s12909-025-07438-7 (PMC12135314; doi:10.1186/s12909-025-07438-7)
Supplement: Supplementary file 2 — Supplementary Material 2 [file 12909_2025_7438_MOESM2_ESM.docx]

| **Table S2. Expert Ratings and Metrics Calculation for Item-Level Content Validity** | | | | | | | | | | | | |
| --- | --- | --- | --- | --- | --- | --- | --- | --- | --- | --- | --- | --- |
| Item | Expert1 | Expert2 | Expert3 | Expert4 | Expert5 | Expert6 | Experts Rated 3/4 | I-CVI | Evaluation | Pc | Kappa, K* | Evaluation |
| Item 1 | 4 | 4 | 4 | 4 | 4 | 3 | 6 | 1.000 | Pass | 0.016 | 1.000 | Excellent |
| Item 2 | 4 | 4 | 3 | 4 | 4 | 3 | 6 | 1.000 | Pass | 0.016 | 1.000 | Excellent |
| Item 3 | 4 | 3 | 3 | 4 | 3 | 3 | 6 | 1.000 | Pass | 0.016 | 1.000 | Excellent |
| Item 4 | 4 | 4 | 3 | 3 | 3 | 2 | 5 | 0.833 | Pass | 0.094 | 0.816 | Excellent |
| Item 5 | 4 | 3 | 3 | 3 | 4 | 4 | 6 | 1.000 | Pass | 0.016 | 1.000 | Excellent |
| Item 6 | 4 | 4 | 4 | 3 | 3 | 3 | 6 | 1.000 | Pass | 0.016 | 1.000 | Excellent |
| Item 7 | 4 | 3 | 3 | 2 | 3 | 3 | 5 | 0.833 | Pass | 0.094 | 0.816 | Excellent |
| Item 8 | 4 | 4 | 4 | 4 | 3 | 3 | 6 | 1.000 | Pass | 0.016 | 1.000 | Excellent |
| Item 9 | 4 | 3 | 3 | 3 | 2 | 4 | 5 | 0.833 | Pass | 0.094 | 0.816 | Excellent |
| Item 10 | 4 | 4 | 4 | 4 | 4 | 3 | 6 | 1.000 | Pass | 0.016 | 1.000 | Excellent |
| Item 11 | 3 | 3 | 3 | 4 | 4 | 4 | 6 | 1.000 | Pass | 0.016 | 1.000 | Excellent |
| Item 12 | 3 | 4 | 4 | 4 | 3 | 3 | 6 | 1.000 | Pass | 0.016 | 1.000 | Excellent |
| Item 13 | 3 | 3 | 3 | 4 | 4 | 3 | 6 | 1.000 | Pass | 0.016 | 1.000 | Excellent |
| Item 14 | 3 | 4 | 4 | 4 | 4 | 3 | 6 | 1.000 | Pass | 0.016 | 1.000 | Excellent |
| Item 15 | 3 | 4 | 4 | 3 | 3 | 4 | 6 | 1.000 | Pass | 0.016 | 1.000 | Excellent |
| Item 16 | 4 | 4 | 4 | 3 | 4 | 4 | 6 | 1.000 | Pass | 0.016 | 1.000 | Excellent |
| Item 17 | 4 | 3 | 3 | 4 | 3 | 4 | 6 | 1.000 | Pass | 0.016 | 1.000 | Excellent |
| Item 18 | 4 | 2 | 3 | 3 | 3 | 4 | 5 | 0.833 | Pass | 0.094 | 0.816 | Excellent |
| Item 19 | 4 | 3 | 3 | 3 | 4 | 4 | 6 | 1.000 | Pass | 0.016 | 1.000 | Excellent |
| Item 20 | 4 | 4 | 3 | 3 | 3 | 3 | 6 | 1.000 | Pass | 0.016 | 1.000 | Excellent |
| Notes: Number of experts: 6, Number of items: 20, Total counts of ratings 3 or 4: 116 | | | | | | | | | | | | |

| **Scale-Level Content Validity (S-CVI)** | | | |
| --- | --- | --- | --- |
| Metric | Value | Threshold | Result |
| S-CVI/UA | 0.80 | ≥0.8 | Pass |
| S-CVI/Ave | 0.967 | ≥0.9 | Pass |

Item-Level Validity (I-CVI): All items met the threshold (I-CVI ≥0.78), with 16/20 items achieving perfect scores (1.00).

Adjusted Kappa values (K*) ranged 0.816–1.000 ("Excellent").

Scale-Level Validity (S-CVI):

S-CVI/UA (0.80) is equal to the 0.80 benchmark, indicating the consensus of the general expert protocol.

S-CVI/Ave (0.967) exceeded the 0.90 threshold, confirming strong overall content validity.
