# Supplementary material for: Status and perceptions of ChatGPT utilization among medical students: a survey-based study
Source: BMC Med Educ. 2025 Jun 4;25:831. doi: 10.1186/s12909-025-07438-7 (PMC12135314; doi:10.1186/s12909-025-07438-7)
Supplement: Supplementary file 3 — Supplementary Material 3 [file 12909_2025_7438_MOESM3_ESM.docx]

**Table S3. Factors Related to Attitude Toward the Use of ChatGPT in medical research in the future**

|  | **What is your attitude towards using ChatGPT for academic research in the future?** | | | | |
| --- | --- | --- | --- | --- | --- |
| **Variable** | **Positive** | **Neutral** | **Negative** | **Cramer's V** | ***P* value^*^** |
| **What do you think are the negative effects of ChatGPT on academic research?** |  |  |  | 0.045 | .764 |
| May spread misinformation | 334 (19.8) | 211 (17.8) | 2 (12.5) |  |  |
| Makes plagiarism easier and harder to detect | 265 (15.7) | 198 (16.7) | 2 (12.5) |  |  |
| May introduce errors or inaccuracies into research texts | 265 (15.7) | 182 (15.3) | 2 (12.5) |  |  |
| Easier to fabricate or falsify research | 243 (14.4) | 155 (13.1) | 2 (12.5) |  |  |
| May introduce bias into literature searches | 145 (8.6) | 99 (8.3) | 1 (6.3) |  |  |
| Makes it more difficult to assess student learning | 138 (8.2) | 117 (9.9) | 2 (12.5) |  |  |
| May introduce bias into research | 117 (6.9) | 92 (7.8) | 1 (6.3) |  |  |
| Increases the imbalance of academic resources | 118 (7.0) | 93 (7.8) | 2 (12.5) |  |  |
| ChatGPT is an expensive or energy-consuming tool | 60 (3.6) | 40 (3.4) | 2 (12.5) |  |  |
| **What specific obstacles did you or your research team encounter?** |  |  |  | 0.066 | .862 |
| Lack of skills or skilled researchers | 93 (22.2) | 45 (23.3) | 0 (0.0) |  |  |
| Lack of usage training tutorials | 93 (22.2) | 45 (23.3) | 0 (0.0) |  |  |
| Don't know how to judge accuracy | 90 (21.5) | 41 (21.2) | 0 (0.0) |  |  |
| Lack of funding | 60 (14.4) | 29 (15.0) | 0 (0.0) |  |  |
| Lack of hardware resources | 75 (17.9) | 30 (15.5) | 1 (100.0) |  |  |
| Others | 7 (1.7) | 3 (1.6) | 0 (0.0) |  |  |

^*^Chi-square *P* value.
